# Consensus strategy in genes prioritization and combined bioinformatics analysis for preeclampsia pathogenesis

**DOI:** 10.1186/s12920-017-0286-x

**Published:** 2017-08-08

**Authors:** Eduardo Tejera, Maykel Cruz-Monteagudo, Germán Burgos, María-Eugenia Sánchez, Aminael Sánchez-Rodríguez, Yunierkis Pérez-Castillo, Fernanda Borges, Maria Natália Dias Soeiro Cordeiro, César Paz-y-Miño, Irene Rebelo

**Affiliations:** 1 Facultad de Medicina, Universidad de Las Américas, Av. de los Granados E12-41y Colimes esq, EC170125 Quito, Ecuador; 2 Department of Molecular and Cellular Pharmacology, Miller School of Medicine and Center for Computational Science, University of Miami, FL 33136 Miami, USA; 3 Department of General Education, West Coast University—Miami Campus, Doral, FL 33178 USA; 4 Departamento de Ciencias Naturales, Universidad Técnica Particular de Loja, Calle París S/N, EC1101608, Loja, Ecuador; 5 Escuela de Ciencias Físicas y Matemáticas, Universidad de Las Américas, Quito, Ecuador; 6 CIQUP/Departamento de Quimica e Bioquimica, Faculdade de Ciências, Universidade do Porto, 4169-007 Porto, Portugal; 7 REQUIMTE, Department of Chemistry and Biochemistry, Faculty of Sciences, University of Porto, 4169-007 Porto, Portugal; 8 Centro de Investigaciones genética y genómica, Facultad de Ciencias de la Salud, Universidad Tecnológica Equinoccial, Quito, Ecuador; 9 Faculty of Pharmacy, University of Porto, Porto, Portugal; 10 UCIBIO@REQUIMTE, Caparica, Portugal

**Keywords:** Consensus analysis, Gene periodization, Preeclampsia, Communality analysis, Microarray analysis, Pathogenesis, Early recognition

## Abstract

**Background:**

Preeclampsia is a multifactorial disease with unknown pathogenesis. Even when recent studies explored this disease using several bioinformatics tools, the main objective was not directed to pathogenesis. Additionally, consensus prioritization was proved to be highly efficient in the recognition of genes-disease association. However, not information is available about the consensus ability to early recognize genes directly involved in pathogenesis. Therefore our aim in this study is to apply several theoretical approaches to explore preeclampsia; specifically those genes directly involved in the pathogenesis.

**Methods:**

We firstly evaluated the consensus between 12 prioritization strategies to early recognize pathogenic genes related to preeclampsia. A communality analysis in the protein-protein interaction network of previously selected genes was done including further enrichment analysis. The enrichment analysis includes metabolic pathways as well as gene ontology. Microarray data was also collected and used in order to confirm our results or as a strategy to weight the previously enriched pathways.

**Results:**

The consensus prioritized gene list was rationally filtered to 476 genes using several criteria. The communality analysis showed an enrichment of communities connected with VEGF-signaling pathway. This pathway is also enriched considering the microarray data. Our result point to VEGF, FLT1 and KDR as relevant pathogenic genes, as well as those connected with NO metabolism.

**Conclusion:**

Our results revealed that consensus strategy improve the detection and initial enrichment of pathogenic genes, at least in preeclampsia condition. Moreover the combination of the first percent of the prioritized genes with protein-protein interaction network followed by communality analysis reduces the gene space. This approach actually identifies well known genes related with pathogenesis. However, genes like HSP90, PAK2, CD247 and others included in the first 1% of the prioritized list need to be further explored in preeclampsia pathogenesis through experimental approaches.

**Electronic supplementary material:**

The online version of this article (doi:10.1186/s12920-017-0286-x) contains supplementary material, which is available to authorized users.

## Background

The study of preeclampsia (PE) from a bioinformatics approach will be affected by several aspects that will inevitable affect the interpretation and will establish an implicit frame to our analysis. The PE is a multifactorial disease that probably involves several genes and environmental factors. However, the main theory behind PE is that the disorder results from placenta ischemia, with further releases of several factors into the maternal circulation [1, 2]. The ischemia origin is supported mainly by a failure in the transformation of the spiral artery caused by a trophoblastic invasion abnormality [1–3]. Therefore, placenta (at this level) is the central organ for pathogenesis. From this point forward, the possible scenarios could be more complex. Nevertheless, the endothelial dysfunction seems to be a primary factor leading to the remaining problems and clinical manifestations. The roll of placenta is clearly reflected in the application of “omic” tools, specifically microarrays studies. The simple inspection of microarray data [4, 5] (through GEO and ArrayExpress databases) reveals that majority were obtained in placenta samples by a case/control design.

Even when arrays technologies could be valuable to provide a wide gene-disease association, a problem arises from the experimental design (case/control). With this type of experimental design will be hard to differentiate pathogenic from non-pathogenic genes. Its means that if we obtain a very significant up or down-regulated gene, we can’t be sure that it is involved in pathogenesis. Moreover we can’t confirm that these up or down-regulated genes can be used as a risk evaluator or predictive measure without further experimental analysis in a longitudinal design. Even with all previous considerations, microarrays information is used for bioinformatics analysis and gene prioritization suggesting that here are genes that can be probably related with pathogenesis [6–12].

How is the scenery in scientific literature? The ratio between case/control and prospective analysis is biased. It is difficult to proof this statement without a rigorous analysis of the scientific information. However, using pre-eclampsia (MeSH Term) in PubMed database, we obtain 13,173 publications in the last 10 years, but adding the terms “longitudinal studies” or “prospective” the previous search is reduced to 1578 in the same time interval. Even when this approach can be considered as superficial clearly indicate the bias toward case/control studies. Therefore, any prioritization strategy based on text mining or even database exploration, will provide us with a genetic-disease association. However we can’t confirm that there genes are primarily related to PE pathogenesis.

There are few studies in PE focused in system biology or other bioinformatics tools [6–14]. Some of them use the microarray information previously described while others used (combined or not), text mining and protein-protein interaction networks (PPI). All these methods will be affected by the previous discussed issues. Still, a more important problem with bioinformatics tools is actually its diversity. There are several ways in which we can combine the information and not all of them will converge into the same results. For example, in the recent work of Miranda van Uitert et al. [6] on microarray data, their proposed several genes but when compared with other two similar studies we found an overlap of 77% with Vaiman et al. [13] and 44% with Moslehi et all [14]. Between these three studies a total of 556 genes were selected but only 47 are common (~8%) which is a very low overlapping (this microarray information will be further discussed).

Each particular problem could have a better tool to solve it and in terms of prioritization, the consensus strategy had proof to be the most effective way to explore gene-disease association [15, 16]. However, we are not so clear if consensus is also effective for identification of pathogenic genes and it will be our first step in the current work. Consequently we will include several prioritization strategies that will be integrated using a consensus strategy in order to rank the genes in the gene-disease association. The consensus result will be integrated in a common pathway and compared with previous results in microarray meta-analysis in order to clarify the genetic function. The goal of this second step including network analysis and metabolic pathways analysis is to additionally evaluate the capacity of identify pathogenic pathways and it relevance.

## Methods

### Selection of pathogenic genes for validation

In order to validate the prioritization strategy on pathogenic genes we need to identify specific genes with a high probability of being involved in PE pathogenesis. Through manually literature inspection we considered a gene as pathogenic if:

1. The silencing or induced overexpression of the proposed gene in animal models generate a clinical phenotype like preeclampsia (this group of genes was named as G1)
2. At least one variation (polymorphism) was associated with PE. We only consider the articles that apply meta-analysis methods (this group of genes was named as G2)

The full analysis of the genes in each group can be found in Additional file 1. We found 35 unique genes combining G1 and G2 groups (off course it is not an exhaustive list). The selected genes in each group and its corresponding Entrez Gene ID identifier are:

G1 (*n* = 27): ADA (100), ADORA2A (135), ADORA2B (136), AGTR1 (185), APOH (350), CD73 (4907), CRP (1401), ENG (2022), EDN1 (1606), FLT1 (2321), GADD45A (1647), HADHA (3030), HIF1A (3091), IDO1 (3620), IL10 (3586), IL17A (3605), IL6 (2569), NOS1 (4842), NOS2 (4843), NOS3 (4846), PGF (5228), ROS1 (6098), TACR3 (6870), TGFB1 (7040), TNF (7124), TNFSF14 (8740), VEGFA (7422).

G2 (*n* = 13): F5 (2153), F2 (2147), AGT (183), MTHFR (4524), NOS3 (4846), ACE (1636), SERPINE1 (5054), VEGFA (7422), LEPR (3953), TGFB1 (7040), AGTR1 (185), HLA-G (3135), IL10 (3586).

### Prioritization algorithms and consensus strategy

From the prioritization portal [15, 16] we selected the methods according to the following criteria: 1) fully available in web service platform and 2) requiring only the disease name for gene prioritization. Under these conditions we found 12 methods: Biograph [17], Candid [18], Glad4U [19], PolySearch [20], Cipher [21], Guildify [22], DisgeNet [23], GeneProspector [24], Genie [25], SNPs3D [26], GeneDistiller [27] and MetaRanker [28]. The following methods: Cipher, Guildify and DisgeNet were not selected from the prioritization portal but from literature, however, fulfilling the same two requirements. These methods have several characteristics that had being fully comprised by other authors previously [15, 29].

Our strategy to combine the different scores obtained in each independent methods is similar to the method used in [30, 31]. Each gene (denoted as i) in the ranked list provided by each method (denoted as j) was normalized (*GeneN*_*i* , *j*_ which means, the normalized score of the gene “i” in the method “j”) in order to integrate all methods for the consensus approach. For the final score by gene, we considered the average normalized score as well as the number of methods which predict the gene (denoted as *n*_*i*_) using the formula:

1 $$ {Gene}_i=\sqrt{\left(\frac{\left({n}_i-1\right)}{\left(12-1\right)}\right)\left(\frac{1}{j}{\varSigma}_j{ Gene N}_{i,j}\right)} $$

The Eq. 1 correspond with the geometrical mean between the average score of each gene obtained in each method and the normalized score according to the number of methods which predict the association between the gene and the disease. However, this formula will be zero if the gene is only predicted by only one method. Therefore we sort the genes according to the *Gene*_*i*_ values and according to the average ($\left[\left(\frac{\left({n}_i-1\right)}{\left(12-1\right)}\right)+\left(\frac{1}{j}{\varSigma}_j\kern0.5em {GeneN}_{i,j}\right)\right]/2$). This sorting will produce a ranking that further normalized leading to the final score of each gene (*ConsenScore*_*i*_). If two genes are predicted by only one method and also have the same normalized scores then will also have the same value of *ConsenScore*_*i*_.

The final list of prioritized genes is actually very long (more than 18,000). We needed a strategy to create a rational cutoff in the number of genes comprising the major pathogenic information with minimal noise. To accomplish this task we used the same pathogenic genes already defined. We defined the following index: ${I}_i=\frac{TP_i}{FP_i+1}{ConsenScore}_i$ where, TP and FP are the true and false positive values (up to the ranking value of the gene i) respectively. The maximal value of *I*_*i*_ can be understood as the maximal compromise between the true positive and false positive rate compensated with the ranking index of each gene.

### Early recognition analysis in prioritization

Several enrichment metrics have been proposed in the chemoinformatics literature to measure the enrichment ability of a virtual screening protocol [32] and had being recently applied in gene prioritization [33]. In this work and similar to [33], we used some of the most extended metrics to estimate the enrichment ability in order to compare different gene prioritization strategies. The overall enrichment metrics include the area under the accumulation curve (*AUAC*); the area under the ROC curve (*ROC*); and the enrichment factor (*EF*) evaluated at the top 1, 5, 10 and 20% of the ranked list. At the same time, the early recognition metrics used were the robust initial enhancement (*RIE*) and the Boltzmann-enhanced discrimination of ROC (*BEDROC*) evaluated at the top 1, 5, 10 and 20% of the ranked list [32]. The calculation of both classic and early recognition enrichment metrics was conducted by using the perl script*Cresset_VS* [34].

### Enrichment analysis

We used David Bioinformatics Resource [35, 36] for gene ontology (GO) and pathways enrichment analysis. The number of GO terms could be very big considering the amount of genes. Therefore we used Revigo [37] in order to simplify the GO terms keeping those with highest specificity. We additionally used RSpider [38], to obtain an integrated pathway combining Reactome and KEGG databases. In these databases the pathways are not the same so any enrichment will produce different pathways that otherwise could be connected or even very similar in the two databases. The use of RSpider will produce not only a statistical analysis of the enrichment but also a network representation integrating the information in both databases. The main goal in RSpider is to connect into non-interrupted sub-network component as many input genes as possible using minimal number of missing genes.

### Protein-protein interaction network and analysis

We used String Database [39] to create the protein-protein interactions network with a confidence cutoff of 0.9 and zero node addition. We also used Cytoscape [40] for centrality indexes calculation and network visualization.

Communality (or cliques) network analysis by clique percolation method was applied using CFinder [41]. The communality analysis provides a better topology description of the network including the location of highly connected sub-graphs (cliques) and/or overlapping modules that usually correspond with relevant biological information. The selection of the value “k-cliques” will affect the number of community and also the number of genes in each community. We create a rational cutoff by balancing the number of communities and the genes distribution across them. In general higher values of k-cliques imply few communities while lower values lead to many communities. In our network both extremes (too small or to high k-cliques values) result in an unbalanced distribution of the genes across communities. Therefore we create the following index “S” as: ${S}^k=\frac{\left| mean\left({N}_g^k\right)- median\left({N}_g^k\right)\right|}{N_c^k}$ where ${N}_g^k$ and ${N}_c^k$ are the number of genes in each community and the number of communities for a defined k-clique cutoff value.

In each community obtained using CFinder, we performed a pathways enrichment analysis followed by a ranking of all pathways. This ranking or scoring was done as follow: if ${ConsenScore}_i^k$ is the *ConsenScore*_*i*_ of the gene “i” in the community “k” then:

1. Each community “k” was weighted as: ${W}_k=\sum {ConsenScore}_i^k/{N}_k$, where *N*_*k*_ is the number of communities.
2. Each pathway “m” was weighted as: ${PathRankScore}_m=\sum {W}_k^m/{N}_k^m$, where ${W}_k^m$ is the weight (*W*_*k*_) of each communities connected with the pathway “m” and ${N}_k^m$ is the number of communities connected with the pathway “m”.
3. A second weight was given to the pathway “m” (*PathGeneScore*_*m*_) considering all the genes involved in the pathway as: ${PathGeneScore}_m=\sqrt{\left\langle {ConsenScore}_i^m\right\rangle \frac{n_m}{N_m}}$, where N_m_ is the total number of genes in the pathway “m” while n_m_ is the number of those genes which are also found in the protein-protein interaction network. $\left\langle {ConsenScore}_i^m\right\rangle$ is the average of the *ConsenScore*_*i*_ of all genes presents in the pathway “m”.
4. The final score associated with the pathway “m” (*PathScore*_*m*_)is calculated as the geometrical mean between *PathGeneScore*_*m*_ and the normalized *PathRankScore*_*m*_.

### Microarray analysis

A total of five studies were considered in microarray data integration and were named as: A1 [7], A2 [6], A3 [14], A4 [8] and A5 [10]. In each study we extracted the significant up-regulated/down-regulated genes considering the procedure of each author in the correspondent articles. In any case was considered the fold-expression as significant cutoff but the adjusted *p*-value reported by the authors. The criterion was an adjusted *p*-value < 0.05. The strategies in the reported articles considering: microarrays integration, gene expression correction and annotations where different (we will discuss more about it in results section and a brief description can be found in Additional file 2). The adjusted *p*-values were used to create a ranking of genes in each study followed by independent normalization.

We could go through a meta-analysis cross-normalization approach as in [10, 33]. However, because different strategies are possible to accomplish this analysis leading to different results we choose to consider each study separately. In each study (“j”) a particular up-regulated or down-regulated gene (“i”) will have a normalized score according to ranking (*GeneS*_*i* , *j*_, the normalized score of the gene “i” in the study “j”). The consensus scoring of each gene in the microarray data was carried out similarly to consensus prioritization strategy. This means, the final score of each gene was calculated as ${GeneAS}_i=\sqrt{\left(\frac{\left({Narray}_i-1\right)}{\left(5-1\right)}\right)\left(\frac{1}{j}{\varSigma}_j{GeneS}_{i,j}\right)}$where, Narray_i_ correspond with the number of studies reporting the gene “i”. Combining all genes in the selected studies we found 1944 genes: 916 always reported up-regulated, 1013 always reported as down-regulated and 13 genes with ambiguous expression. The full list of genes is presented in Additional file 2 as well as the calculated scores. This final score (*GeneAS*_*i*_) will have a double meaning 1) inter-studies agreement and 2) the measure of the statistical significance of the gene in the study. Therefore, highest values of the score imply that the gene was identifying in several studies and also with highest statistical significance.

## Results

### Consensus prioritization

The detections of pathogenic genes in all methods are presented in Table 1. As we can notice not all methods are capable to identify the 35 proposed pathogenic genes.

**Table 1:** Identification (in %) of pathogenic genes in each approach

Methods	1%			5%			10%			20%		
	G1	G2	G1,2	G1	G2	G1,2	G1	G2	G1,2	G1	G2	G1,2
BioCarta	0,00	0,00	0,00	0,00	7,69	2,86	3,70	23,08	8,57	3,70	23,08	8,57
Candid	14,81	15,38	14,29	25,93	61,54	34,29	29,63	69,23	37,14	44,44	84,62	54,29
GLAUG4	3,70	0,00	2,86	14,81	15,38	14,29	18,52	53,85	28,57	22,22	76,92	37,14
PlySearch	0,00	0,00	0,00	0,00	7,69	6,25	6,25	15,38	12,50	12,50	23,08	18,75
CIPHRE	0,00	0,00	0,00	0,00	7,69	2,86	0,00	15,38	5,71	0,00	15,38	5,71
Guildify	14,81	23,08	14,29	18,52	38,46	22,86	25,93	53,85	34,29	44,44	69,23	51,43
DISGENET	3,70	15,38	5,71	7,41	30,77	14,29	11,11	38,46	20,00	29,63	92,31	45,71
Geneprospector	3,70	30,77	11,43	22,22	84,62	34,29	22,22	100,00	40,00	33,33	100,00	48,57
GENIE	7,41	0,00	5,71	14,81	46,15	25,71	25,93	76,92	40,00	37,04	84,62	48,57
SPNS3D	7,41	7,69	5,71	14,81	15,38	11,43	14,81	30,77	17,14	29,63	53,85	34,29
GeneDistiller	0,00	0,00	0,00	0,00	7,69	6,25	6,25	7,69	12,50	12,50	15,38	18,75
MetaRanker	44,44	92,31	54,29	62,96	92,31	68,57	74,07	92,31	77,14	88,89	92,31	88,57
Consensus	51,85	100,00	62,86	74,07	100,00	80,00	85,19	100,00	88,57	88,89	100,00	91,43

Consensus strategy identify the entire G2 set in the first 1% of the final gene list (>18,000 genes) and in all cases remain as the method with higher identification of pathogenic genes. Very close to consensus strategy approach is the MetaRanker [28] method. The identification of the pathogenic genes is important but even more relevant is the early recognition ability.

The average rank of the studied genes is lower in consensus strategy than in other methods (Table 2) used independently. The average rank of the detected genes is not properly speaking a measure of early recognition. However, intuitively will means that consensus strategy early detect the pathogenic genes (identified in G1 and G2 groups). The MetaRanker is one more time the closer strategy. Even when these two previous analyses could indicate that the consensus strategy prioritize better the pathogenic genes, we additionally calculate several indexes directly related with the evaluation of early enrichment (Table 3). Because MetaRanker is the method with closer results, the early enrichment analysis was only performed comparing consensus and MetaRanker strategies.

**Table 2:** Average rank of identified pathogenic genes in each method

Methods	1%			5%			10%			20%		
	G1	G2	G1,2	G1	G2	G1,2	G1	G2	G1,2	G1	G2	G1,2
BioCarta					4,0	4,0	7,0	5,7	5,7	7,0	5,7	5,7
Candid	18,8	9,0	17,2	44,1	70,9	58,3	71,3	92,0	73,8	180,7	157,9	182,5
GLAUG4	1		1	3,0	5,0	3,2	4,8	8,3	6,4	6,5	11,2	9,1
PlySearch	0	0	0		1,0	1,0	2,0	1,5	1,5	3,0	2,3	2,3
CIPHRE					15,0	15,0		28,5	28,5		28,5	28,5
Guildify	36,0	41,0	29,0	57,0	155,6	117,6	300,1	324,0	353,9	1010,9	648,9	864,1
DISGENET	2,0	1,5	1,5	3,0	2,8	3,0	6,3	4,6	5,7	15,5	13,4	13,8
Geneprospector	4,0	2,5	2,5	11,2	7,7	7,7	11,2	9,8	9,6	24,0	9,8	16,7
GENIE	2,0		2,0	5,8	7,7	6,4	11,0	12,3	10,9	19,2	13,8	15,8
SPNS3D	2,0	3,0	2,0	4,0	5,5	4,0	4,0	8,0	6,2	12,9	13,4	13,9
GeneDistiller	0,0	0,0	0,0		1,0	1,0	1,0	1,0	1,0	1,5	1,5	1,3
MetaRanker	45,8	43,7	44,9	143,8	43,7	114,5	297,3	43,7	231,4	648,6	43,7	511,9
Consensus	36,4	15,8	28,0	118,2	15,8	88,3	272,6	15,8	205,7	372,0	15,8	282,4

**Table 3:** Initial enrichment indexes for the MetaRanker and the Consensus strategy

Indexes	MetaRanker			Consensus		
	G1	G2	G1,2	G1	G2	G1,2
MEDIAN RANK	246 (6–12,345)	23 (3–3709)	154 (3–12,345)	114 (2–9218)	10 (1–59)	46 (1–9218)
AUC	0,927	0,982	0,938	0,944	1	0,957
EF_1%	44,533	92,491	54,394	51,993	100,272	63,028
EF_5%	12,604	18,478	13,726	14,823	20,011	16,009
EF_10%	7,41	9234	7717	8519	10	8857
EF_20%	4445	4616	4429	4444	5	4571
RIE_1%	38,836	75,295	46,407	48,1	92,346	58,435
RIE_5%	11,807	17,656	12,941	13,806	19,673	15,158
RIE_10%	6912	9117	7324	7676	9918	8191
RIE_20%	3971	4731	4,11	4216	5013	4399
BEDROC_1%	0,418	0,78	0,51	0,517	0,956	0,642
BEDROC_5%	0,599	0,889	0,66	0,7	0,991	0,772
BEDROC_10%	0,696	0,915	0,739	0,773	0,995	0,827
BEDROC_20%	0,79	0,941	0,819	0,84	0,998	0,877

The indexes related with the early enrichment clearly state that consensus strategy over perform the result of MetaRanker in pathogenic genes detection locating more genes with a significant lower rank. We compare the rank of the pathogenic genes between the two methods for G1, G2 and G1,2 using signed Wilconson test. The *p*-value was lower than 0.01 in the three groups indicating statically significant differences in the ranking obtained by the two methods.

Previous calculations are based on predefined genes in G1 and G2 groups. In order to explore the consistency of our results by changing those genes, we performed a bootstrap sampling as follow:

- We remove 5 random genes form the 35 “pathogenic genes” (around 14%) and evaluate the median rank of the remaining ones in both: consensus and MetaRanker
- We repeat the previous step 1000 times, each time selecting a new set of 5 random genes.

**Fig. 1:**
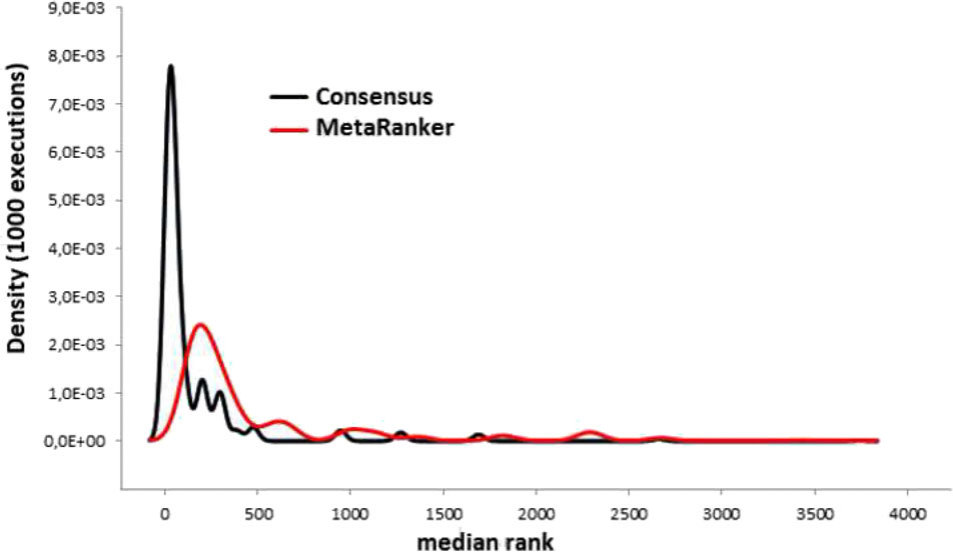
Average ranking distribution in consensus and MetaRanker strategies in 1000 generations randomly removing the 14% of the pathogenic genes (G1,2) each time

The density distribution (using Gaussian kernel of function “density” in R) of the 1000 values in both methods is presented in the Fig. 1.

As we can noticed, consensus strategies compared to MetaRanker provides more frequently the lower rank for the genes in agreement with our previous results in Table 3 after evaluation in 1000 samples modifications.

### Enrichment analysis of preeclampsia related genes and protein-protein interaction network

**Fig. 2:**
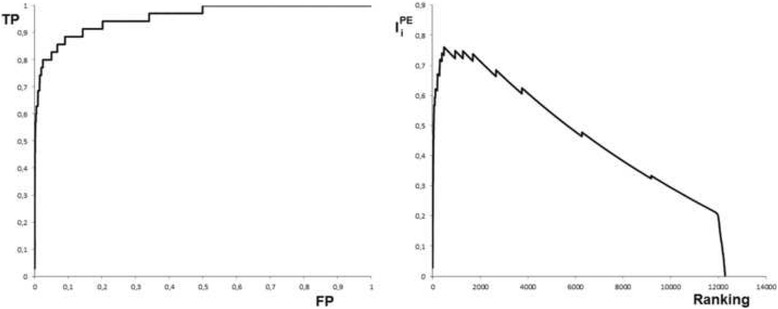
Left) ROC curve obtained with prioritized genes for PE and the proposed pathogenic list. Right) Variation of *I*_*i*_ with respect to genes ranking. The maximal value of *I*_*i*_ is the 0.76085 and correspond with a ranking value of 476

Using G1 and G2 unique genes (*n* = 35), we can notice (Table 1) that consensus strategy already identify the 89% in the 10% of the data, this means that the 89% of the 35 genes are in the initial 1800 genes obtained from prioritization. This is a very big number; therefore a strategy for a rational cutoff was designed (see Methods). The implementation of I_i_ considering the true positive and false positive ratio could be used as a rational cutoff to reduce the amount of genes. This procedure is showed in Fig. 2.

The maximal (Fig. 2) value of *I*_*i*_ is 0.76085 and correspond with a ranking value of 476, therefore the reduced list for PE comprise the first 476 genes. The entire gene list as well as their scores and ranking can be found in Additional file 3. In the 476 genes there are 30 of 35 predefined pathogenic genes.

The enrichment analysis of biological processes in these genes results into more than 500 terms with an adjusted *p*-value <0.01 (considering FDR) (Additional file 4). In order to simplify this list we used Revigo [37] to calculate the frequencies of the gene ontology terms. We only consider those terms with a frequency lower than 0.01% (full list of terms can be found in Additional file 4). With this consideration the number of terms remains high so only some of the initial ones (more relevant) are presented in Table 4.

**Table 4:** Some of the more specific biological process obtained by enrichment analysis in PE genes

BP ID	Name	Frequency	log10 *p*-value
GO:0008217	regulation of blood pressure	0,01%	−33,0101
GO:0032496	response to lipopolysaccharide	0,01%	−16,8268
GO:0030193	regulation of blood coagulation	0,01%	−16,684
GO:0050818	regulation of coagulation	0,01%	−16,6635
GO:0043434	response to peptide hormone	0,01%	−15,6819
GO:0048660	regulation of smooth muscle cell proliferation	0,00%	−15,3242
GO:0032868	response to insulin	0,01%	−13,5114
GO:0045765	regulation of angiogenesis	0,01%	−13,1612
GO:0070663	regulation of leukocyte proliferation	0,01%	−12,3862
GO:0031960	response to corticosteroid	0,00%	−12,214
GO:0045428	regulation of nitric oxide biosynthetic process	0,00%	−11,8447
GO:0043627	response to estrogen	0,01%	−11,8447
GO:0050670	regulation of lymphocyte proliferation	0,01%	−11,3468
GO:0007568	aging	0,01%	−11,3468
GO:0050730	regulation of peptidyl-tyrosine phosphorylation	0,01%	−11,1844
GO:0003073	regulation of systemic arterial blood pressure	0,01%	−11,0177
GO:0051384	response to glucocorticoid	0,00%	−10,9101
GO:0010743	regulation of macrophage derived foam cell differentiation	0,00%	−10,8962
GO:0050729	positive regulation of inflammatory response	0,01%	−10,8962
GO:0050886	endocrine process	0,00%	−10
GO:0031099	regeneration	0,01%	−9,9245
GO:0051341	regulation of oxidoreductase activity	0,00%	−9,8996
GO:0019229	regulation of vasoconstriction	0,00%	−9,8761
GO:0042035	regulation of cytokine biosynthetic process	0,01%	−9284
GO:0019218	regulation of steroid metabolic process	0,01%	−8,1221

Similarly, the enrichment analysis of metabolic pathways is presented in Table 5 using to main databases: KEGG and Reactome.

**Table 5:** Pathways enrichment analysis using Reactome and KEGG databases

Pathway Name (KEGG)	% Genes	*p*-value
Cytokine-cytokine receptor interaction	16,0337553	1,22E-26
Complement and coagulation cascades	7,59,493,671	1,64E-20
Graft-versus-host disease	4,85,232,068	1,33E-13
Allograft rejection	4,21,940,928	1,28E-10
Focal adhesion	9,28,270,042	4,28E-09
Type I diabetes mellitus	4,21,940,928	4,58E-09
Antigen processing and presentation	5,69,620,253	1,25E-08
Hematopoietic cell lineage	5,48,523,207	1,95E-07
Jak-STAT signaling pathway	7,17,299,578	1,91E-06
Renin-angiotensin system	2,32,067,511	2,63E-05
TGF-beta signaling pathway	4,64,135,021	2,13E-04
Adipocytokine signaling pathway	4,00843882	2,91E-04
Endocytosis	6,96,202,532	5,37E-04
Natural killer cell mediated cytotoxicity	5,69,620,253	6,70E-04
Pathway Name (Reactome)	% Genes	FDR
Hemostasis	15,8,227,848	7,21E-29
Signaling in Immune system	12,2,362,869	7,53E-11
Signaling by PDGF	5,06329114	1,73E-08
Signaling by VEGF	2,10,970,464	2,00E-06
Integrin cell surface interactions	4,85,232,068	2,05E-05

The pathways presented in Table 5 are only a partial list but it is entirely presented for Reactome and KEGG in Additional file 4 *.*

The biological processes and enriched pathways are consistent between them and also with the scientific knowledge about PE, however would be hard to establish some kind of relevance between them without further consideration. In this way we carried on a network analysis.

**Fig. 3:**
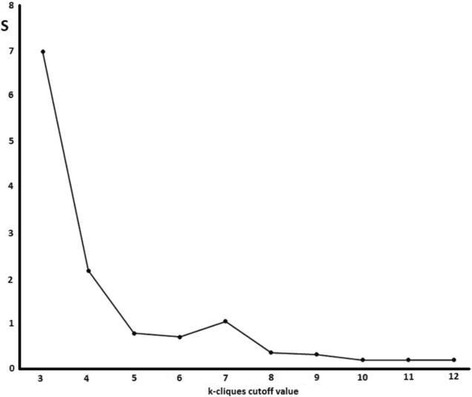
Values of *S* ^*k*^ with respect to each k-clique cutoff value

**Fig. 4:**
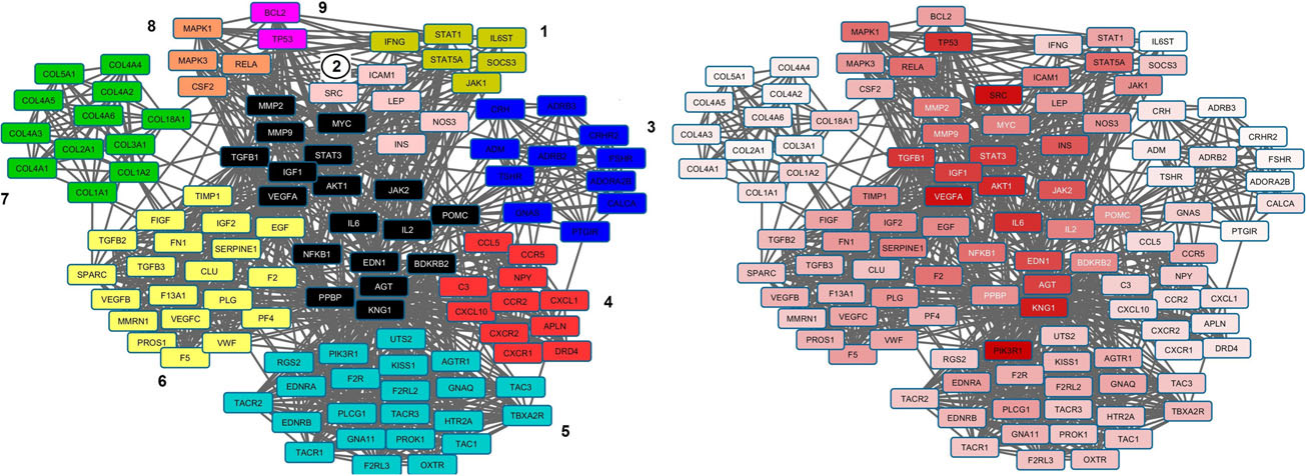
*Left*). Community analysis for k-cliques = 9. *Black* nodes represent genes which are parts of several communities. The rest of the colors correspond with the 9 communities obtained. *Right*) Gradient connectivity degree distribution (min = 9 with *white* color and max = 85 with *red* color and indicated by PIK3R1 gene)

With the indicated cutoff of 0.9, the final protein-protein interaction network has 417 nodes, corresponding with the 87.6% of the initial genes (476). The *S* ^*k*^ index (as proposed in the Methods section to identify a rational k-clique number) will rich a minimum either by an increment in the number of communities and/or by an increment in the similarity between the mean and median values of the number of genes in all communities. We can notice (Fig. 3) that the desired values will be between 8 and 10. The number of communities for k = 8 is 16 compared to 9 and 5 for k = 9 and 10 respectively. Considering that in each community several biological analyses will be carried on, 16 communities will be difficult to study. Additionally in k = 8, one of the communities have almost twofold the number of genes with respect the remaining communities. For this reason we select the k = 9 in our analysis (Fig. 4. Left).

Each community can be weighted considering the *ConsenScore*_*i*_of each gene in the community (see Table 6). Additionally we also included the number of pathogenic genes present in the community.

**Table 6:** Communities membership and scores

Communities	Genes	Average *ConsenScore*_*i*_	Average Rank	Average Degree	N Pathogenic	N Pathways
2	TGFB1, SRC,IGF1,IL6, INS, LEP, NOS3, AKT1, ICAM1, MMP2, STAT3, VEGFA, EDN1, MMP9	0,995	89,07	55.21	5	8
6	TGFB1, TGFB3, EGF, VWF, IGF1, F2, KNG1, PPBP, SERPINE1, TIMP1, FN1, PLG, VEGFA, IGF2, CLU, F13A1, FIGF, MMRN1, PF4, SPARC, VEGFB, VEGFC, F5, TGFB2, PROS1	0,991	171,28	38.24	5	7
9	TGFB1, IL6, NFKB1, TP53, AKT1, MMP2, BCL2, MYC, MMP9	0,989	194,56	52.33	2	6
1	IL6, IL2, STAT3, IFNG, STAT5A, JAK2, JAK1, SOCS3, STAT1, IL6ST	0,989	210,2	39.60	1	4
8	MAPK1, MAPK3, NFKB1, IL2, STAT3, RELA, CSF2, JAK2, MYC	0,989	210,67	44.89	0	14
5	AGT, AGTR1, BDKRB2, EDNRA, F2R, F2RL2, F2RL3, GNA11, GNAQ, KNG1, OXTR, PIK3R1, PLCG1, PROK1, RGS2, TAC1, TAC3, TACR1, TACR3, UTS2, EDN1, EDNRB, HTR2A, KISS1, TACR2, TBXA2R	0,987	231,04	33.88	4	7
3	ADM, CRH, POMC, ADORA2B, PTGIR, TSHR, ADRB2, ADRB3, CALCA, CRHR2, GNAS, FSHR	0,987	243,92	16.50	1	3
4	POMC, CCR5, AGT, APLN, BDKRB2, C3, CCL5, CCR2, CXCL10, CXCR1, CXCR2, DRD4, IL8, KNG1, NPY, PPBP, CXCL1	0,985	273,25	28.75	1	5
7	COL18A1, COL1A1, COL1A2, COL3A1, COL4A5, COL2A1, COL4A1, COL4A2, COL4A6, COL5A1, COL4A3, COL4A4	0,983	304,5	15.58	0	2

Communities 2 and 6 could be considered as the more relevant showing. However could be useful the prioritization of the metabolic pathways by an enrichment analysis in each community (*full list presented in* Additional file 4) and weighted as presented in the Methods section.

### Microarray data integration

**Fig. 5:**
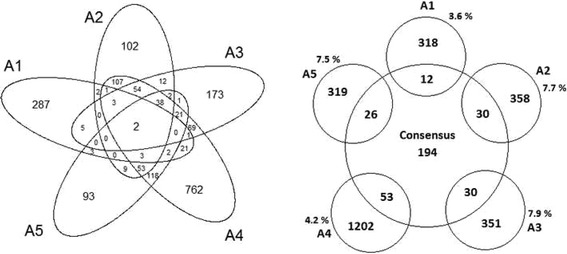
*Left*) Venn diagrams between the five microarray studies. *Right*) Agreement between each microarray study and the consensus gene list

From the 1944 genes collected in microarrays data only 80 are present in our 476 obtained in consensus prioritization representing only a 4%. The worst gene overlapping with respect to other microarrays is with the study A1. The A2, A3, A4, A5 conserve 40 genes in common but drastically reduced to 2 by adding A1 (Fig. 5 Left). The agreement between selected microarray studies is not good in terms of genes identifications as we can see in the Venn diagrams (Fig. 5, Left). It is a direct consequence of the differences in initial microarray data and processing strategies (presented in Additional file 2). The study A1 is the only one with any meta-analysis strategy. Both A2 and A3 carried out a meta-analysis, while A4 and A5 go specifically through cross-platform normalization. The differences between both strategies in microarray data integration had being explored previously [42]. Actually A2 and A3 share 111 genes and similarly A4 and A5 share 237 genes. This gene space is reduced 40 genes when all four studies are combined. Moreover as we can see in Additional file 2, A4 and A5 share a number of similarities regarding initial microarray data.

Analyzing the amount of genes that each study independently shares with the initial 476 prioritized genes (Fig. 5. Right) we found that: A1 (*n* = 12, 3.8%), A2 (*n* = 30, 7.7%), A3 (*n* = 30, 7.9%), A4 (*n* = 53, 4.2%) and A5 (*n* = 26, 7.5%). The result indicates that the methodology of Moslehi et al. [14] will represent better our prioritized genes (even when very close to A2 and A5). Moreover considering the average consensus score of those genes we found: A1 (0.396), A2 (0.561), A3 (0.597), A4 (0.111) and A5 (0.540). This average scoring also suggests that the work of Moslehi et al. [14] also cover better ranked genes (even not so distant of A2 and A5). These values will be discussed later.

There are a total of 41 up-regulated and 39 down-regulated genes commonly found between all integrated genes in microarray data and the 476 already prioritized genes (a total of 80 genes). The up-regulated are: VEGFA, FLT1, STOX1, SERPINE1, LEP, INHA, INHBA, ENG, HMOX1, VWF, TGFB1, TFPI, ADAM12, CRH, PAPPA2, VEGFC, CP, MMP14, FN1, SERPINA3, SIGLEC6, ACE2, PREP, FABP4, EGFR, FSTL3, IL6ST, VDR, IGFBP5, MMP15, ITGA5, TRIM24, CGA, MET, DUSP1, MIF, TAPBP, NR1H2, MMP11, HPN, GLRX and the down-regulated are: ACVRL1, ADRB3, AGTR1, ANGPT1, CD4, CD14, COL1A1, COL1A2, F5, F13A1, FCER1G, FGF2, GHR, CFH, HGF, HSD11B2, CFI, IGF1, IGFBP7, IL10RA, IDO1, JAK1, KLRD1, MMP1, NEDD4, ENPP1, PLAUR, MAPK1, CCL2, SOD1, SPP1, TGFBR3, THBS1, TLR4, VCAM1, APLN, HGS, ROCK2, PLAC1. From these 80 genes 34 (42.5%) were located with a ranking less than 180 (around the first 1% of the list) in the consensus strategy prioritization.

**Fig. 6:**
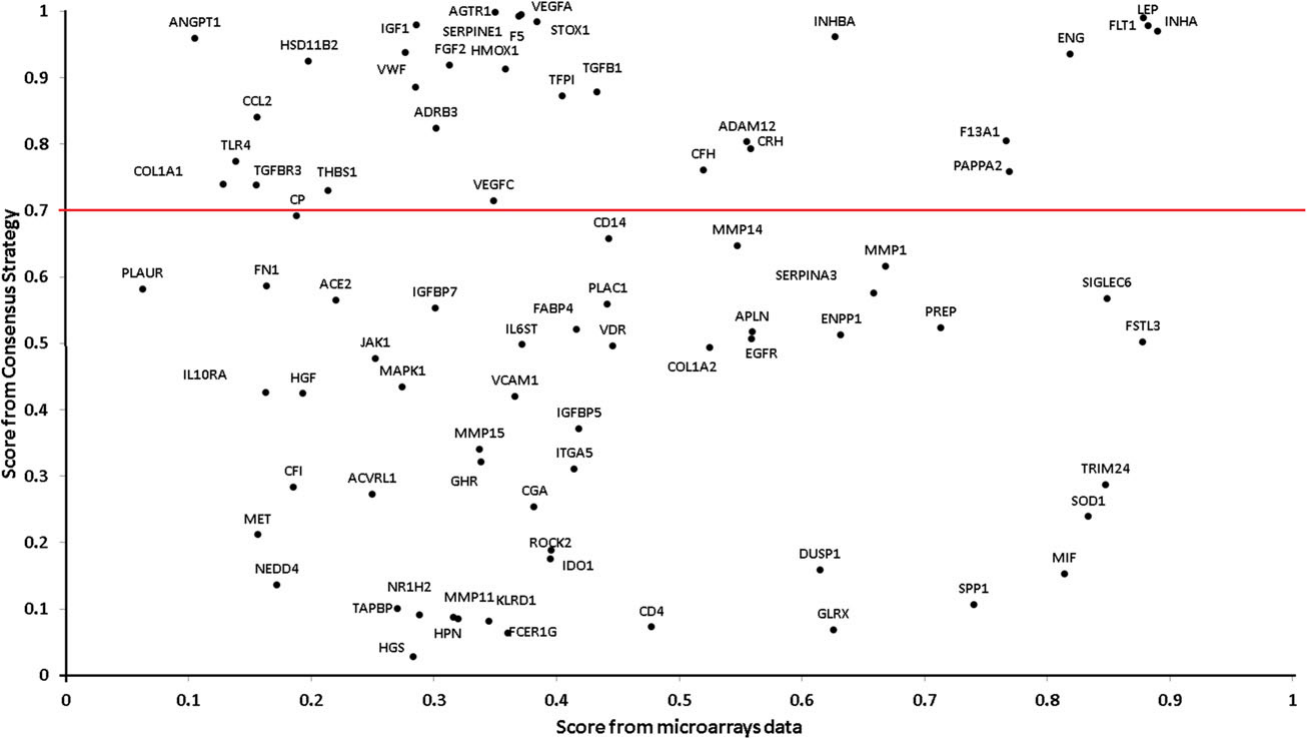
Relationship between the score obtained from microarray data and the consensual strategy prioritization. The red line indicates and scores in consensus prioritization equal to 0.7

Comparing the scores of consensus strategy and scores obtained from the microarrays studies (Fig. 6) we can arrive to some interesting results. Moreover, from these 80 genes 72 are also present in the 417 forming the interaction network and 19 are also part of some community. We can evaluate the contribution of these 19 genes in each community using the average *GeneAS*_*i*_ of the genes which belong to a particular community in a similar way as we did previously (Table 6). The corresponding weights for each community are: 1 (0.062), 2(0.140), 3(0.072), 4(0.033), 5(0.014), 6(0.132), 7(0.054), 8(0.031) and 9(0.048). These weights also confirm that communities 2 and 6 could be the more relevant as previously presented.

### Integrated metabolic network

Using RSpider [38] from the 476 genes only 272 were mapped to a reference global network obtaining three significant models (Table 8).

**Fig. 7:**
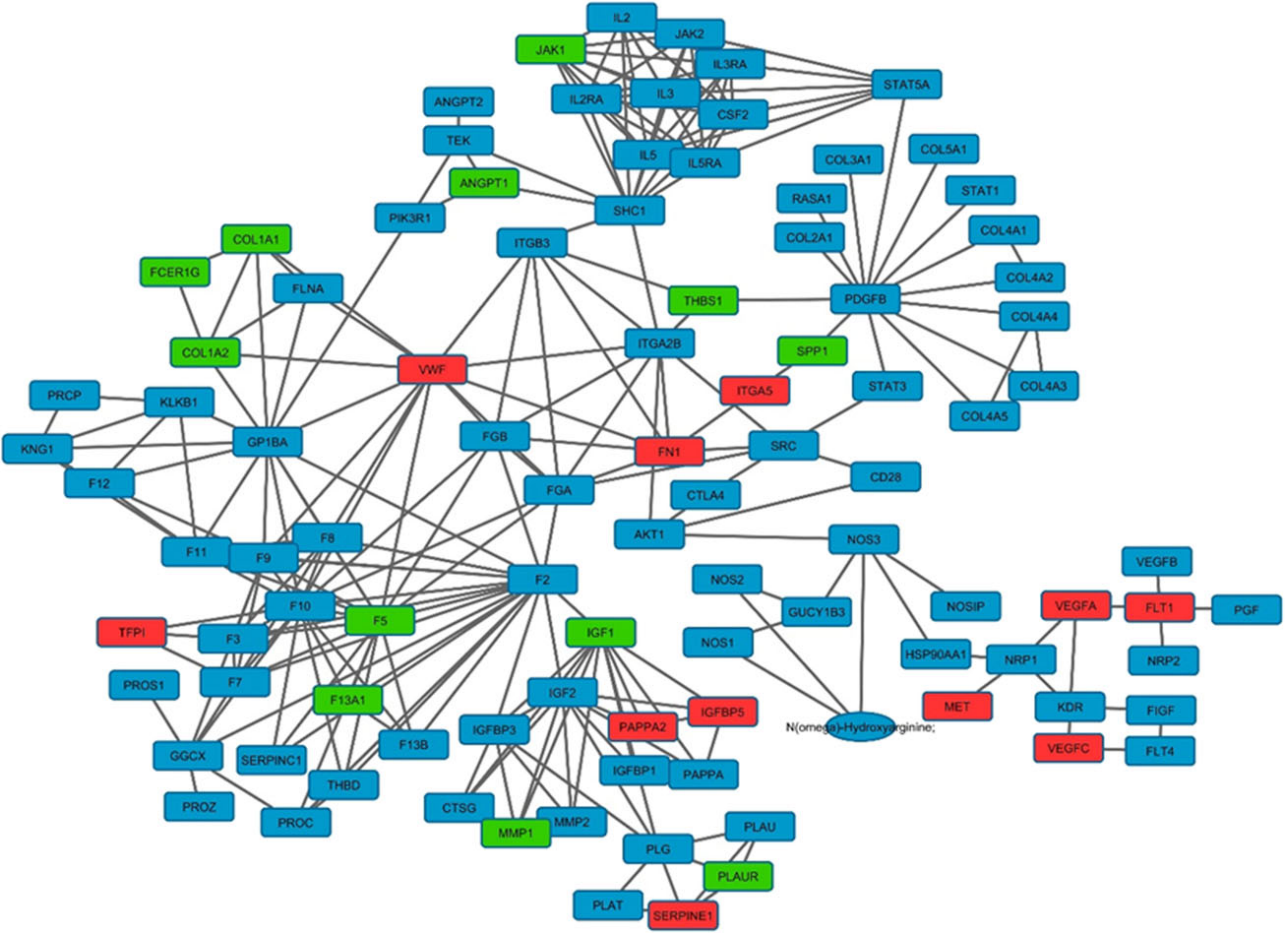
Integrated metabolic network with 98 genes colored according to our microarray data. The color are: *green*, *red* and *blue*, indicating down-regulated, up-regulated and no information from microarray respectively

The *p*-value indicates the probability for a random gene/protein list to have a maximal connected component of the same or larger size. This *p*-value is computed by Monte Carlo simulation as described in [38]. Beside this statistical analysis, we should also consider that in the initial data (476) there are 80 genes also matching with microarray data while in the smallest network 23 of 98 are also present in the microarray information. This enrichment is statistically different (*p*-value = 0.036) compared to random gene extraction. The 23 genes are: ANGPT1, COL1A1, COL1A2, F5, F13A1, FCER1G, FLT1, FN1, IGF1, IGFBP5, ITGA5, JAK1, MET, MMP1, SERPINE1, PLAUR, SPP1, TFPI, THBS1, VEGFA, VEGFC, VWF and PAPPA2. The network associated with Model 1 is presented in Fig. 7. The network in Model 3 of Table 8 is presented in Additional file 5.

The expanded integrated metabolic network (Model 3) allows the entrance of 114 genes in order to bring connections between initial genes. However, it also incorporates 32 compounds that also act as connectors. These compounds obtained from the integrated network are presented in Table 8, some of them could be very generic like “fatty acids” o could be very specifics as “serotonin” or “L-Homocysteine”. These compounds can be easily grouped mainly in lipids, steroids, amino acids, and purine metabolisms and will be further explored in the discussion.

## Discussion

### Consensus prioritization and enrichment analysis

Our results confirm that the consensus strategy actually improve the detection and prioritization of pathogenic genes. Application of early recognition measures are important and should be considered together with identification capabilities. The ability to rank the relevant genes on the top of a long prioritized list will directly reduce de cost of experimental validation. Previous authors had being probed that consensual strategy in prioritization improve the detection of genes related with specific pathology [15, 16, 33]. However, we are proving here for the first time that the consensus strategy also improves the early enrichment ability of genes related with pathogenesis (at least in PE).

Any study in gene-disease association is intrinsically focused into pathogenesis discovery. During this process obviously some relations could be established and not necessarily because of pathogenesis but a secondary modification (the experimental design will be directly related with this type of result). If several prioritization strategies are combined, then, the possibility of removing noisy relationships (in pathogenesis terms) increases as well as the agreement in relevant genes.

The biological processes as well as metabolic pathways enrichment analysis lead us to already expected information. Some of the biological processes, like those related to blood pressure or vasoconstrictions have a direct association with PE clinical development. Biological processes associated with inflammation, angiogenesis, cytokine, immune system and hormones regulation could also be associated with PE clinical manifestation or even pathogenesis [6, 7, 43, 44] and there are also well related with the metabolic pathways enrichment results (Table 5). The pathway analysis also reflects a good agreement with previous works. The cytokine pathway, VEGF and PDGF signaling, immune system and even some of the cancer related pathways were previously reported by other authors [6, 7, 9, 14, 44, 45]. Signaling pathways in general, are highly relevant as well as several routes connected with cancer (*see* Additional file 4) which also agree with the previous studies [14, 46, 47].

### Protein-protein interaction network, communality analysis and microarrays integration

The enrichment analysis can be helpful. However it is hard to establish a ranking of the pathways according to their implications in pathogenesis without further analysis. It is the main reason to combine the analysis of the protein-protein interaction network. The entire network contains 417 nodes but only 111 are part of some community. The network with 417 already comprises 29 of the 35 predefined pathogenic genes. The sub-network containing only genes which belong to some community have 12 of 29 predefined pathogenic genes. Moreover only 3 genes (HADHA, IDO1 and HLA-G) of the remaining 17 genes are not directly connected with the sub-network. On the other hand, the average degree of the pathogenic genes is 23.6 which is statistically significant higher than non-pathogenic genes (14.2) at *p*-value <0.05. This result indicates that the node degree could be associated with pathogenesis in this network. The black colored nodes represent those genes that are present in more than one community and therefore are usually those with higher connectivity degree as we can also see in the Fig. 3 Right (and Table 6).

The top 20 genes with highest connectivity degree are: PIK3R1, SRC, VEGFA, KNG1, AKT1, IL6, TP53, TGFB1, STAT3, IGF1, AGT, EDN1, JAK2, INS, EGFR, SHC1, MAPK8, MMP9, STAT5A and MAPK1. The majority of them are located between communities (black colored) and only 3 (EGFR, SHC1 and MAPK8) were not identified as member of any community. The community’s analysis (Table 6) indicates that communities 2 and 6 could be considered as the more relevant showing: 1) the highest scores; 2) the minimal average ranking and 3) both include the major number of pathogenic genes. In terms of connectivity degree the community 2 have the greater value instead community 6 which have a middle one. Looking at genes in the community 2 we can clearly identify elements of the VEGF signaling and also NOS metabolism through AKT1 and generally a core of possible mechanism well established in PE that will be discussed later. Moreover, the prioritization of the metabolic pathways shown that VEGF signaling pathway is not only the most relevant pathway (Table 7) but also it is exclusively enriched in community 2.

**Table 7:** Pathways enrichment analysis in communities and their associated weights

Pathways	*PathRankScore*_*k*_	N Community	*PathGeneScore*_*m*_	*PathScore*_*m*_	Community
VEGF signaling pathway	89,07	1	0,422	0,547	2
mTOR signaling pathway	130,18	2	0,445	0,505	2,6
Adipocytokine signaling pathway	169,98	3	0,518	0,479	1,2,8
Intestinal immune network for IgA production	171,28	1	0,497	0,467	6
Leukocyte transendothelial migration	89,07	1	0,303	0,463	2
Progesterone-mediated oocyte maturation	89,07	1	0,301	0,462	2
Cytokine-cytokine receptor interaction	185,95	4	0,529	0,455	1,2,4,6
Jak-STAT signaling pathway	176,12	4	0,468	0,445	1,2,8,9
Renin-angiotensin system	231,04	1	0,804	0,443	5
MAPK signaling pathway	149,87	2	0,378	0,439	8,9
Complement and coagulation cascades	225,19	3	0,708	0,432	4,5,6
TGF-beta signaling pathway	190,97	2	0,486	0,427	6,8
Focal adhesion	188,28	3	0,464	0,422	2,6,7
Apoptosis	194,56	1	0,431	0,396	9
Regulation of actin cytoskeleton	171,28	1	0,326	0,378	6
Natural killer cell mediated cytotoxicity	210,67	1	0,453	0,375	8
ErbB signaling pathway	210,67	1	0,451	0,374	8
Fc epsilon RI signaling pathway	210,67	1	0,450	0,374	8
T cell receptor signaling pathway	210,67	1	0,436	0,368	8
Neurotrophin signaling pathway	202,61	2	0,339	0,338	8,9
Toll-like receptor signaling pathway	226,16	3	0,432	0,335	4,8,9
NOD-like receptor signaling pathway	241,96	2	0,510	0,326	4,8
Dorso-ventral axis formation	210,67	1	0,331	0,321	8
B cell receptor signaling pathway	210,67	1	0,328	0,319	8
Cell cycle	194,56	1	0,252	0,303	9
Chemokine signaling pathway	231,37	3	0,370	0,300	1,4,8
Gap junction	231,04	1	0,351	0,293	5
Calcium signaling pathway	237,48	2	0,363	0,284	3,5
Neuroactive ligand-receptor interaction	237,48	2	0,343	0,276	3,5
Vascular smooth muscle contraction	237,48	2	0,327	0,270	3,5
Melanogenesis	231,04	1	0,281	0,262	5
ECM-receptor interaction	304,50	1	0,478	0,040	7

Actually we can notice that tops pathways primarily involve communities 2 and 6. It indicates that those communities as well as their genes are highly relevant in PE. Additionally, the community number 5 is exclusively related with the Renin-angiotensin system and considering that it is also enriched in neuroactive ligand-receptor interaction and vascular smooth muscle contraction, we can suspect that this community has a strong connection with the hypertensive disorder. Interestingly community 8 have the major number of associated pathways. However, most of them are related with signaling pathways like TGF-beta signaling. The enrichment in signaling processes could indicate that it is probably a central group of genes acting as connectors between several metabolic processes and therefore would be relevant to comprehend PE heterogeneity.

Following the importance of community 2 and 6, the major pathways ordered by relevance connected to both communities are: VEGF signaling pathway, mTOR signaling pathway, Adipocytokine signaling pathway, Intestinal immune network for IgA production, Leukocyte transendothelial migration, Progesterone-mediated oocyte maturation, Cytokine-cytokine receptor interaction, Jak-STAT signaling pathway, complement and coagulation cascades, TGF-beta signaling pathway, focal adhesion and regulation of actin cytoskeleton.

In order to explore our results using additional experimental information we included the microarrays analysis. The worst genes overlapping with respect to prioritized list is with the study A1 (Fig. 5), while the other four studies show more consistent results. The reason for this difference in A1 is mainly because is the only study that is not a meta-analysis. We included it because it is the largest independent study.

Our result indicates that the study of Moslehi et al. [14] identify more common genes and also better ranked in our consensus strategy. Both studies using meta-analysis (A2 and A3) shown a better agreement with consensus than A4 and A5. The A5 is better that A4, and similar to A2 and A3 probably because the use of *combat* [48] and an increased number of arrays. Also, A2 is the study that carried out the largest microarray integration related with PE. The use of *combat* in cross-platform normalization had being favored in term of clinical and biological meaning agreement [42]. The differences in the percentage of genes (Fig. 5 Righ) shared with consensus strategy is really small comparing A3 with A2 and A5 studies. The A3 study also comprise some similarities with A4 and A5 regarding initial microarray data. However, A3 study exclusively considers meta-analysis in microarrays of early-onset preeclampsia. It is a very important difference regarding other studies. We had probed that consensus prioritization actually improve pathogenic early recognition and we also known that genes involved in early onset preeclampsia are probably closer to pathogenesis than late-onset preeclampsia. This could explain why the A3 have the highest average score with our prioritized consensus list. Therefore it is a logical result considering the previous analysis and also an indirect validation of our consensus strategy. The A5 and A4, consider similar microarrays than A3 but including other do not exclusively related with early-onset preeclampsia.

Regarding all differences between microarrays studies, we should remember that all genes extracted from microarray data were statistically significant up or down-regulated in each corresponding study. Moreover we carried out a complete integration of the gene space between all microarrays data considered, so, to our effects we didn’t exclude any genes because be part or not of a particular study. Therefore, this disparity in microarrays studies could only affect the *GeneAS*_*i*_ scoring. The score should be interpreted as a commitment between agreement across methods and their statistical significance. Even when is reasonable to assume that a gene with a simultaneously high agreement and high statistical significance could be very important (i.e. LEP, FLT1, INHA) (Fig. 6), also the condition of high agreement with low statistically significance is equally relevant (because actually leads to highest score). In other words, a statistical significance don’t necessarily means that the gene is more relevant to the disease than any other with a reduced but significant change.

Previously we presented evidence indicating that consensus prioritization is capable to identify genes with high pathogenic probabilities in the first portion of the data. It is clearly presented in Fig. 6 with VEGFA, AGTR1, F5 and TGFB1 which are well related with pathogenesis [49–55]; however, the score obtained from microarray data is relatively low in these cases (less than 0.5). Considering a high cutoff value (i.e. >0.7) we can identify: LEP, FLT1, INHA, ENG, PAPPA2, and CRH. There are sufficient evidences to associate these genes with PE pathogenesis or clinical manifestation [14, 52, 53, 56–60]. Our calculations also indicate that communities 6 and 2 are those with highest enrichment of genes coming from microarray data confirming our previous results using the network and consensus prioritization (Table 6). This consistency support that the prioritization strategy is actually pointing us in the correct direction and also justify the idea that the associated pathways could also be highly relevant.

### Metabolic involvement

In all previous analysis, the VEGF signaling pathways had being selected as the most relevant in PE. This pathway is presented (Fig. 6) with the genes: VEGFA, VEGFB, VEGFC, FLT1, KDR, FLT4, PGF, NRP2 and NRP1. These genes are directly connected with arginine (NOS1, NOS2 and NOS3) and nitric oxide metabolisms (NOSIP and HSP90AA1). Considering the previous results in communities and pathways enrichment analysis we should conclude that this processes will be actually the most significant for PE pathogenesis. Interestingly the involvement of VEGF, FLT1 and several elements in arginine metabolism, including NO production, was proposed in [61] as the primary mechanism in placenta leading to PE.

In the protein-protein interaction network analysis we can noticed that genes like VEGFA, NOS3, SRC and AKT1 are highly connected (community 2 in Table 6 and Fig. 4) but in the integrated metabolic network (Model 1, Fig. 7) their connectivity is not so elevated (or even in the extended integrated metabolic network showed in Additional file 5). The reason for these differences is a direct consequent of the pathway representation in KEGG and Reactome. For example, VEGF mediates the ezrin/calpain/PI3K/Akt pathway-dependent stimulation of NOS3 phosphorylation leading to Ca2+ independent NO generation [62–64]. This connection will be reflected in the PPI as an edge between VEGFA and NOS3 or even between VEGFA and PIK3R1 (distant nodes in Fig. 6) and these both interactions will not be seen in the Fig. 7. This is why we should additionally use the Model 3 for integrated pathway (presented in Additional file 5) and consider the pathway in Fig. 7 as the simplest representation of the biological meaning of genes involved in PE. Form the metabolic integrated network (Fig. 6) we can clearly identify some well-known mechanisms related with VEGF and PE and other relevant effects that will be discussed further.

The increment in FLT1 production (noticed by the microarray data) could lead to the increment in the soluble-Flt1 rescuing the extracellular VEGFA [65, 66]. Therefore the increment in VEGFA expression in placenta could be a compensatory response to restore normal angiogenesis [67]. This mechanism of interaction between soluble-Flt1 and VEGFA as well as soluble-Eng and TGFB1 (also present in community 2) had being long term related with PE pathogenesis [66]. The increment in VEGFA could also be associated with an increment in HSP90, also acting with SRC in the NOS3 expression and NO production. As we previously explained, this can also be accomplished through PI3K and the involvement of AKT1. The expression of HSP90 could be polemic because apparently it is related with the disease progression and also placenta location [68]. However several authors had being found an increment in the placenta expression of HSP90 in PE [68–70] at mRNA and protein levels. Moreover, this increment can be a protective reaction that is stimulated by HIF1A [71] and consequently connected to disease progression as described in [68]. Actually, there are differences in HIF1A placenta expression in early-onset PE and late-onset PE [72] showing that both stages have different hypoxia compensatory mechanism. Interestingly the HIF1A gene is part of our prioritized list (ranking at 46) and PPI network. HIF1A is not part of any community but connected to several of them, especially with community 2 (HIF1A is not connected with communities 1 and 7) and it is also missing in the integrated metabolic models. There are not studies of HSP90 (or closely related HSP70) gene variations or promoter polymorphism in PE to know for sure if the protective roll could be compromised leading to early or late PE manifestation.

In the extended model (Model 3, in Additional file 5) the VEGF pathways is connected with several members of the HLA family through CD247 and PAK2 but also to another variety of pathways through ROCK2 and FGD3. Both connections can be related with apoptosis, trophoblastic affectation and endothelial cells organization [73, 74]. Even when PAK2 had being poorly studied in PE, we know that it is directly involved in gestational trophoblastic disease [45] and that endothelial cells PAK and/or CDC42 are directly involved with KDR and consequently essential for endothelial cells organization [74, 75].

The roll of angiogenesis in PE pathogenesis is clearly revealed in our theoretical analysis as well as scientific literature. However, we should discuss other aspects related with pathogenesis, specially the renin-angiotensin pathway and the roll of catechol-O-methyltransferase (COMT).

We can notice in our prioritization list that AGT and AGTR1 are the first two genes in our ranking (ACE and AGTR2 were also found in position 7 and 19 respectively) but interestingly only AGTR1 was found down-regulated in our microarray data. The down-regulation of AGTR1 from microarray data was only identified (considering our microarray data) in [6, 8], however, other authors [76, 77] found an increment in AGTR1 expression. In any case there are evidences that renin-angiotensin system is modified in hypoxic conditions [77, 78] and it is well connected with the HIF1A previously discussed. In our list of pathogenic genes, we should notice the AGT derive from 1) the AT_1_-AA auto-antibody that interact with AGTR1 or 2) from some polymorphism in AGT that had being associated with increased risk of preeclampsia [54, 79, 80]. The origin of AT_1_-AA in PE is quite unknown [50, 51, 81] but some authors shown that it is related to B-cells and it is connected with IL10 during pregnancy as well as with other cytokines (i.e. TNF) [82, 83]. Other authors indicates that an increment in CD4(+) T-cells and a decrement in T regulatory cells stimulates TNF, IL6, endothelin (EDN1), IL-17 and B-cells production of AT_1_-AA [83–85]. Some of these genes are clearly involved in our networks and communities, especially EDN1 and IL6. We can’t clearly state that renin-angiotensin pathway is not part of PE pathogenesis but our network based results reduce the importance of this pathway. These suggest that its finding in the top prioritized gene list is actually a consequence of the hypertension effect more than the PE pathogenesis.

The additional consideration of the compounds involved in the integrated metabolic network (Model 3) (Table 8) also leads us to interesting points. A deregulation in ammonia and urea cycles as well as in phospholipid and bile acid metabolism has being reported previously in metabolomics analysis [86, 87]. We know that steroid hormones are related with vascular endothelium, for instance, estradiol and progesterone/estradiol ratios are altered in placenta of PE women probably related with NO metabolism [88]. However, one of the most relevant results in this Table 9 and in the expanded integrated metabolic network (Model 3) is the presence of catechol-O-methyltransferase (COMT) and 2-methoxyoestradiol. In our prioritized gene list (see Additional file 3) the COMT gene is ranked at position 47 and we know that pregnant mouse with a deficiency in catechol-O-methyltransferase (COMT) (consequently no 2-methoxyoestradiol) lead to PE phenotype [89]. This animal model was not considered in our initial pathogenic data analysis but it is clearly expressed in the integrated pathway and prioritization strategy. In the model 3 (Additional file 5), the COMT gene is quite far connected with VEGF, however, was recently showed that 2-methoxyestradiol has an anti-angiogenic effect connected to KDR and HIF1A probably through a different mechanism not involving sFlt-1 [90].

**Table 8:** Compound list of metabolic species present in the expanded integrated metabolic network model

3-Oxopalmitoyl-CoA	C05259	Deoxyadenosine	C00559
Corticosterone	C02140	Deoxyinosine	C05512
Arachidonate	C00219	Tetrahydrofolate (THF)	C00101
3alpha,7alpha-Dihydroxy-5beta-cholestanoyl-CoA	C04644	Bromobenzene-3,4-oxide	C14839
11beta-Hydroxyandrost-4-ene-3,17-dione	C05284	Parathion (DNTP)	C06604
3alpha,7alpha-Dihydroxy-5beta-cholestanate;	C04554	Oxitriptan	C00643
2-Methoxy-17beta-estradiol	C05302	Bilirubin	C00486
Triglyceride	C00422	Docosahexaenoic acid (DHA)	C06429
5(S)-HPETE	C05356	Nicotinamide mononucleotide (NMN)	C00455
16alpha-Hydroxyestrone	C05300	dAMP	C00360
Prostaglandin G2	C05956	Melatonin	C01598
L-Homocysteine	C00155	Nicotinamide	C00153
Glutathione (GSH)	C00051	Serotonin	C00780
Serine	C00065	N(omega)-Hydroxyarginine;	C05933

**Table 9:** Results of integrated metabolic pathways

Models	Number of initial genes	*p*-value
Model 1 (0 missing gene(s) are allowed)	98	< 0.005
Model 2 (1 missing gene(s) are allowed)	209	< 0.005
Model 3 (2 missing gene(s) are allowed)	231	< 0.005

Our results confirm that consensus prioritization strategy lead us to genes with pathogenic involvement, at least in PE. Moreover, the introductions of network and enrichment analysis are capable to narrow the metabolic and gene space leading us toward reasonable conclusions in agreement with our scientific knowledge of the disease. However, the proposed strategies need to be further improved in several topics. For instance: a) the inclusion of prioritization algorithms based in learning strategies, b) the inclusion of other network processing methods to reduce the gene lost and 3) the differentiation between early and late-onset preeclampsia. Additionally, as previously stated, there are several genes relevant in our analysis with poor or almost no information in their PE involvement. Therefore further experimental analysis will needed to validate the participation of these genes in PE pathogenesis or clinical manifestation.

## Conclusions

From all the prioritization methods used in our work MetaRanker brings the better results. However, our results confirm that consensus strategy of several prioritization tools improve the detection and initial enrichment of pathogenic genes, at least in preeclampsia condition.

The combination of around the first percent of the prioritized genes and protein-protein interaction network followed by communality analysis brings the possibilities to reduce the gene space and actually group well known genes related with pathogenesis. In this analysis communities connected with VEGF-signaling pathway are highly enriched. This pathway is also enriched considering the microarray data. Actually the pathways weighting strategy together with network analysis agrees with the results obtained in microarray data.

The integrated metabolic pathway clearly indicates main routes involved in preeclampsia pathogenesis. Our result could support previous publications indicating that hypoxia and also angiotensin pathways are secondary manifestations and could be actually connected with disease progression or differentiation between early and late onset preeclampsia development. Our result point to VEGF, FLT1, KDR as relevant pathogenic genes, as well as those connected with NO metabolism. However, other genes like HSP90, PAK2, CD247 and others included in the first 1% of the prioritized list need to be further explored in preeclampsia pathogenesis through experimental approaches.

## Additional files

Additional file 1: Identification of pathogenic genes. The file comprises the literature and several observations considered for the selection of our pathogenic gene list. (DOCX 86 kb)

Additional file 2: Microarrays consensus. The file comprises all information concerning the microarray data as well as the integration. (XLSX 141 kb)

Additional file 3: Prioritized genes. The file comprises our final prioritized genes as well as the consensus score. (XLSX 28 kb)

Additional file 4: Enrichment analysis. The file comprises all the enrichment analysis: gene ontology and metabolic pathways. (XLSX 292 kb)

Additional file 5: Integrated metabolic network. The file comprises the Integrated Metabolic Network corresponding with Model 3 of Table 8 as well as the list of all compounds contained in the metabolic network. (DOCX 1116 kb)

## Abbreviations

AUAC: Under the accumulation curve
BEDROC: Boltzmann-enhanced discrimination of ROC
EF: Enrichment factor
PE: Preeclampsia
RIE: Robust initial enhancement (RIE)
ROC: Receiver operating curve
