# Supplementary material for: Consensus strategy in genes prioritization and combined bioinformatics analysis for preeclampsia pathogenesis
Source: BMC Med Genomics. 2017 Aug 8;10:50. doi: 10.1186/s12920-017-0286-x (PMC5549357; doi:10.1186/s12920-017-0286-x)
Supplement: Supplementary file 1 — Identification of pathogenic genes. The file comprises the literature and several observations considered for the selection of our pathogenic gene list. (DOCX 86 kb) [file 12920_2017_286_MOESM1_ESM.docx]

**Supplementary Materials 1**

***Identification of pathogenic genes***

**Group 1. Animal Models**

| **Primary**  **Gene** | **Secondary**  **Genes** | **References** | **Observations** |
| --- | --- | --- | --- |
| ADA | ADORA2A, ADORA2B, CD73 (NT5E), FLT1 | [1] |  |
| HIF1A | TNFSF14, FLT1, AGTR1 | [2] |  |
| TNFSF14 | TNFSF14, FLT1, AGTR1 |  |  |
| AGTR1 | TNFSF14, FLT1, AGTR1 |  | These three studies used AT1-AA auto-antibody to induce the PE like manifestations. |
| AGTR1 | IL6, ET1 (EDN1), FLT1, ENG, TGFB1, | [3] |  |
| AGTR1 |  | [4], [5] |  |
| TNF | FLT1, AT1-AA, ENG | [5] |  |
| GAD45A | P38 MAPK (family 11-14) | [6] |  |
| IL6 |  | [7] |  |
| IL17A | ROS1 | [8] |  |
| CRP | TACR3 | [9] |  |
| FLT1 | VEGFA, PGF, ET1 (EDN1) | [10], [11] |  |
| PGF |  | [10], [12] |  |
| ENG | TGFB1 | [13] |  |
| APOH | HADHA | [14] |  |
| TNF | HIF1A, TLR3, TLR4 | [15] |  |
| IDO1 |  | [16] |  |
| NOS1,NOS2,NOS3 |  | [17], [18] |  |

Notes

The “primary gene” is the target of the stimulus. The “secondary genes” are genes that are also modified by the initial stimulus, therefore, could be also related with the pathogenesis. The only condition that needs to be further discussed is the AT1-AA. The pathogenesis following this model start with the presence of the AT1-AA antibody which secondarily affect the AGTR1, therefore, it is not the AGT or AGT1 the primary modification but actually a consequence of the still unknown origin of AT1-AA

*Final List*

N=27.

ADA, ADORA2A, ADORA2B, AGTR1, APOH, CD73, CRP, ENG, EDN1, FLT1, GADD45A, HADHA, HIF1A, IDO1, IL10, IL17A, IL6, NOS1, NOS2, NOS3, PGF, ROS1, TACR3, TGFB1, TNF, TNFSF14, VEGFA,

***Group 2 (Gene Polymorphisms through meta-analysis studies)***

| **Gene** | **Associations/Reference** | **Gene** | **Associations/Reference** |
| --- | --- | --- | --- |
| MMP9 | - [19], - [20] | CX3CR1 | - [21] |
| HLA-G | +*[22]; - [23] | SELE | - [21] |
| MTHFR | + [24]; - [21]; + [25]; + [26]; - [23]; +* [27] | GNB3 | - [21] |
| IL10 | +* [28]; - [29]; - [21]; - [23] | CYBA | - [21] |
| TGFB1 | - [21]; +[30] | SELP | - [21] |
| F5 | + [21]; + [31]; + [23] | RGS2 | - [21] |
| F2 | + [21] ; + [23] | ADIPOQ | - [21] |
| LEPR | + [21] ; - [23] | APOE | - [21]; - [23] |
| CD28 | - [21] | COMT | - [21] |
| CTLA4 | - [21]; - [23] | CYP 17A1 | - [21] |
| ICOS | - [21] | DIO1 | - [21] |
| IFNG | - [21] | LEP | - [21]; - [23] |
| IL6 | - [21]; - [23] | DBH | - [21] |
| ICAM1 | - [21] | SCNN1B | - [21] |
| KIR3DL2 | - [21] | ESR1 | - [21] |
| MBL2 | - [21] | FAS | - [21] |
| TNF | - [21]; - [23] | SOD3 | - [21] |
| CYP11B2 | - [21] | VEGFA | + [32]; +[33]; - [23] |
| ACE | - [21] ; +* [23]; + [34]; +* [35] | SERPINA1 | + [36]; + [37]; - [23] |
| AGT | - [21] ; +* [23]; + [38]; +[39] | NOS3 | + [40]; + [41]; - [23]; +* [42] |
| ANGPT1 | - [21] | AGTR1 | + [43]; - [21] ; +* [23]; -[44] |
| PPARG | - [23] | EPHX1 | - [23] |
| THBD | - [23] |  |  |

*Notes*

Association: (+), (-) indicates that the study found at least one polymorphism with positive or null association respectively. The (+*) notation indicates that some polymorphisms were positive associates in at least one type of population.

*Final list*

We selected only genes with at least one (+*). The final list is (N=13)

F5, F2, AGT, MTHFR, NOS3, ACE, SERPINE1, VEGFA, LEPR, TGFB1, AGTR1, HLA-G, IL10.

*References*

[1] T. Iriyama, K. Sun, N. F. Parchim, J. Li, C. Zhao, A. Song, L. A. Hart, S. C. Blackwell, B. M. Sibai, L.-N. L. Chan, T.-S. Chan, M. J. Hicks, M. R. Blackburn, R. E. Kellems, and Y. Xia, “Elevated placental adenosine signaling contributes to the pathogenesis of preeclampsia.,” *Circulation*, vol. 131, no. 8, pp. 730–41, Feb. 2015.

[2] T. Iriyama, W. Wang, N. F. Parchim, A. Song, S. C. Blackwell, B. M. Sibai, R. E. Kellems, and Y. Xia, “Hypoxia-independent upregulation of placental hypoxia inducible factor-1α gene expression contributes to the pathogenesis of preeclampsia.,” *Hypertension*, vol. 65, no. 6, pp. 1307–15, Jun. 2015.

[3] Y. Xia and R. E. Kellems, “Angiotensin receptor agonistic autoantibodies and hypertension: preeclampsia and beyond.,” *Circ. Res.*, vol. 113, no. 1, pp. 78–87, Jun. 2013.

[4] R. Dechend, P. Gratze, G. Wallukat, E. Shagdarsuren, R. Plehm, J.-H. Bräsen, A. Fiebeler, W. Schneider, S. Caluwaerts, L. Vercruysse, R. Pijnenborg, F. C. Luft, and D. N. Müller, “Agonistic autoantibodies to the AT1 receptor in a transgenic rat model of preeclampsia.,” *Hypertension*, vol. 45, no. 4, pp. 742–6, Apr. 2005.

[5] M. R. Parrish, S. R. Murphy, S. Rutland, K. Wallace, K. Wenzel, G. Wallukat, S. Keiser, L. F. Ray, R. Dechend, J. N. Martin, J. P. Granger, and B. LaMarca, “The effect of immune factors, tumor necrosis factor-alpha, and agonistic autoantibodies to the angiotensin II type I receptor on soluble fms-like tyrosine-1 and soluble endoglin production in response to hypertension during pregnancy.,” *Am. J. Hypertens.*, vol. 23, no. 8, pp. 911–6, Aug. 2010.

[6] X. Liu, Q. Deng, X. Luo, Y. Chen, N. Shan, and H. Qi, “Oxidative stress-induced Gadd45α inhibits trophoblast invasion and increases sFlt1/sEng secretions via p38 MAPK involving in the pathology of pre-eclampsia.,” *J. Matern. Fetal. Neonatal Med.*, pp. 1–10, Mar. 2016.

[7] B. Lamarca, J. Speed, L. F. Ray, K. Cockrell, G. Wallukat, R. Dechend, and J. Granger, “Hypertension in response to IL-6 during pregnancy: role of AT1-receptor activation.,” *Int. J. Interf. cytokine Mediat. Res.*, vol. 2011, no. 3, pp. 65–70, Nov. 2011.

[8] P. Dhillion, K. Wallace, F. Herse, J. Scott, G. Wallukat, J. Heath, J. Mosely, J. N. Martin, R. Dechend, and B. LaMarca, “IL-17-mediated oxidative stress is an important stimulator of AT1-AA and hypertension during pregnancy.,” *Am. J. Physiol. Regul. Integr. Comp. Physiol.*, vol. 303, no. 4, pp. R353–8, Aug. 2012.

[9] N. F. Parchim, W. Wang, T. Iriyama, O. A. Ashimi, A. H. Siddiqui, S. Blackwell, B. Sibai, R. E. Kellems, and Y. Xia, “Neurokinin 3 receptor and phosphocholine transferase: missing factors for pathogenesis of C-reactive protein in preeclampsia.,” *Hypertension*, vol. 65, no. 2, pp. 430–9, Feb. 2015.

[10] S. E. Maynard, J.-Y. Min, J. Merchan, K.-H. Lim, J. Li, S. Mondal, T. A. Libermann, J. P. Morgan, F. W. Sellke, I. E. Stillman, F. H. Epstein, V. P. Sukhatme, and S. A. Karumanchi, “Excess placental soluble fms-like tyrosine kinase 1 (sFlt1) may contribute to endothelial dysfunction, hypertension, and proteinuria in preeclampsia.,” *J. Clin. Invest.*, vol. 111, no. 5, pp. 649–58, Mar. 2003.

[11] S. R. Murphy, B. B. D. LaMarca, K. Cockrell, and J. P. Granger, “Role of endothelin in mediating soluble fms-like tyrosine kinase 1-induced hypertension in pregnant rats.,” *Hypertension*, vol. 55, no. 2, pp. 394–8, Feb. 2010.

[12] H. Suzuki, A. Ohkuchi, S. Matsubara, Y. Takei, M. Murakami, M. Shibuya, M. Suzuki, and Y. Sato, “Effect of recombinant placental growth factor 2 on hypertension induced by full-length mouse soluble fms-like tyrosine kinase 1 adenoviral vector in pregnant mice.,” *Hypertension*, vol. 54, no. 5, pp. 1129–35, Nov. 2009.

[13] S. Venkatesha, M. Toporsian, C. Lam, J. Hanai, T. Mammoto, Y. M. Kim, Y. Bdolah, K.-H. Lim, H.-T. Yuan, T. A. Libermann, I. E. Stillman, D. Roberts, P. A. D’Amore, F. H. Epstein, F. W. Sellke, R. Romero, V. P. Sukhatme, M. Letarte, and S. A. Karumanchi, “Soluble endoglin contributes to the pathogenesis of preeclampsia.,” *Nat. Med.*, vol. 12, no. 6, pp. 642–9, Jun. 2006.

[14] X. Ding, Z. Yang, Y. Han, and H. Yu, “Long-chain fatty acid oxidation changes in a β2 glycoprotein I-induced preeclampsia-like mouse model.,” *Placenta*, vol. 35, no. 6, pp. 392–7, Jun. 2014.

[15] G. Bobek, L. Surmon, K. M. Mirabito, A. Makris, and A. Hennessy, “Placental Regulation of Inflammation and Hypoxia after TNF-α Infusion in Mice.,” *Am. J. Reprod. Immunol.*, vol. 74, no. 5, pp. 407–18, Nov. 2015.

[16] M. K. Santillan, C. J. Pelham, P. Ketsawatsomkron, D. A. Santillan, D. R. Davis, E. J. Devor, K. N. Gibson-Corley, S. M. Scroggins, J. L. Grobe, B. Yang, S. K. Hunter, and C. D. Sigmund, “Pregnant mice lacking indoleamine 2,3-dioxygenase exhibit preeclampsia phenotypes.,” *Physiol. Rep.*, vol. 3, no. 1, Jan. 2015.

[17] S. V Ramesar, I. Mackraj, P. Gathiram, and J. Moodley, “Sildenafil citrate decreases sFlt-1 and sEng in pregnant l-NAME treated Sprague-Dawley rats.,” *Eur. J. Obstet. Gynecol. Reprod. Biol.*, vol. 157, no. 2, pp. 136–40, Aug. 2011.

[18] S. Baijnath, N. Soobryan, I. Mackraj, P. Gathiram, and J. Moodley, “The optimization of a chronic nitric oxide synthase (NOS) inhibition model of pre-eclampsia by evaluating physiological changes.,” *Eur. J. Obstet. Gynecol. Reprod. Biol.*, vol. 182, pp. 71–5, Nov. 2014.

[19] C. M. Wang and S. L. Zhang, “Non-association of MMP-9 -1562C/T polymorphism with preeclampsia risk: evidence from a meta-analysis.,” *Clin. Exp. Obstet. Gynecol.*, vol. 42, no. 6, pp. 730–5, 2015.

[20] L.-L. Gong, H. Liu, and L.-H. Liu, “Lack of association between matrix metalloproteinase-9 gene-1562C/T polymorphism and preeclampsia: a meta-analysis.,” *Hypertens. pregnancy*, vol. 33, no. 4, pp. 389–94, Nov. 2014.

[21] F. M. Fong, M. K. Sahemey, G. Hamedi, R. Eyitayo, D. Yates, V. Kuan, S. Thangaratinam, and R. T. Walton, “Maternal genotype and severe preeclampsia: a HuGE review.,” *Am. J. Epidemiol.*, vol. 180, no. 4, pp. 335–45, Aug. 2014.

[22] N. Pabalan, H. Jarjanazi, C. Sun, and A. C. Iversen, “Meta-analysis of the human leukocyte antigen-G (HLA-G) 14 bp insertion/deletion polymorphism as a risk factor for preeclampsia.,” *Tissue Antigens*, vol. 86, no. 3, pp. 186–94, Sep. 2015.

[23] E. Staines-Urias, M. C. Paez, P. Doyle, F. Dudbridge, N. C. Serrano, J. P. A. Ioannidis, B. J. Keating, A. D. Hingorani, and J. P. Casas, “Genetic association studies in pre-eclampsia: systematic meta-analyses and field synopsis.,” *Int. J. Epidemiol.*, vol. 41, no. 6, pp. 1764–75, Dec. 2012.

[24] X. Wu, K. Yang, X. Tang, Y. Sa, R. Zhou, J. Liu, Y. Luo, and W. Tang, “Folate metabolism gene polymorphisms MTHFR C677T and A1298C and risk for preeclampsia: a meta-analysis.,” *J. Assist. Reprod. Genet.*, vol. 32, no. 5, pp. 797–805, May 2015.

[25] X. Li, Y. L. Luo, Q. H. Zhang, C. Mao, X. W. Wang, S. Liu, and Q. Chen, “Methylenetetrahydrofolate reductase gene C677T, A1298C polymorphisms and pre-eclampsia risk: a meta-analysis.,” *Mol. Biol. Rep.*, vol. 41, no. 8, pp. 5435–48, Aug. 2014.

[26] X. Wang, H. Wu, and X. Qiu, “Methylenetetrahydrofolate reductase (MTHFR) gene C677T polymorphism and risk of preeclampsia: an updated meta-analysis based on 51 studies.,” *Arch. Med. Res.*, vol. 44, no. 3, pp. 159–68, Apr. 2013.

[27] X. Xia, W. Chang, and Y. Cao, “Meta-analysis of the methylenetetrahydrofolate reductase C677T polymorphism and susceptibility to pre-eclampsia.,” *Hypertens. Res.*, vol. 35, no. 12, pp. 1129–34, Dec. 2012.

[28] W. Yang, Z. Zhu, J. Wang, W. Ye, and Y. Ding, “Evaluation of association of maternal IL-10 polymorphisms with risk of preeclampsia by A meta-analysis.,” *J. Cell. Mol. Med.*, vol. 18, no. 12, pp. 2466–77, Dec. 2014.

[29] Y. H. Lee, J.-H. Kim, and G. G. Song, “Meta-analysis of associations between interleukin-10 polymorphisms and susceptibility to pre-eclampsia.,” *Eur. J. Obstet. Gynecol. Reprod. Biol.*, vol. 182, pp. 202–7, Nov. 2014.

[30] X. Li, L. Shen, and H. Tan, “Polymorphisms and plasma level of transforming growth factor-Beta 1 and risk for preeclampsia: a systematic review.,” *PLoS One*, vol. 9, no. 5, p. e97230, 2014.

[31] X. Wang, T. Bai, S. Liu, H. Pan, and B. Wang, “Association between thrombophilia gene polymorphisms and preeclampsia: a meta-analysis.,” *PLoS One*, vol. 9, no. 6, p. e100789, 2014.

[32] D. Cheng, Y. Hao, W. Zhou, and Y. Ma, “Vascular endothelial growth factor +936C/T, -634G/C, -2578C/A, and -1154G/A polymorphisms with risk of preeclampsia: a meta-analysis.,” *PLoS One*, vol. 8, no. 11, p. e78173, 2013.

[33] G. G. Song, J.-H. Kim, and Y. H. Lee, “Associations between vascular endothelial growth factor gene polymorphisms and pre-eclampsia susceptibility: a meta-analysis.,” *Immunol. Invest.*, vol. 42, no. 8, pp. 749–62, 2013.

[34] W. G. Zhong, Y. Wang, H. Zhu, and X. Zhao, “Meta analysis of angiotensin-converting enzyme I/D polymorphism as a risk factor for preeclampsia in Chinese women.,” *Genet. Mol. Res.*, vol. 11, no. 3, pp. 2268–76, 2012.

[35] Z. Chen, F. Xu, Y. Wei, F. Liu, and H. Qi, “Angiotensin converting enzyme insertion/deletion polymorphism and risk of pregnancy hypertensive disorders: a meta-analysis.,” *J. Renin. Angiotensin. Aldosterone. Syst.*, vol. 13, no. 1, pp. 184–95, Mar. 2012.

[36] J. A. Morgan, S. Bombell, and W. McGuire, “Association of plasminogen activator inhibitor-type 1 (-675 4G/5G) polymorphism with pre-eclampsia: systematic review.,” *PLoS One*, vol. 8, no. 2, p. e56907, 2013.

[37] L. Zhao, M. B. Bracken, A. T. Dewan, and S. Chen, “Association between the SERPINE1 (PAI-1) 4G/5G insertion/deletion promoter polymorphism (rs1799889) and pre-eclampsia: a systematic review and meta-analysis.,” *Mol. Hum. Reprod.*, vol. 19, no. 3, pp. 136–43, Mar. 2013.

[38] R. Lin, Y. Lei, Z. Yuan, H. Ju, and D. Li, “Angiotensinogen gene M235T and T174M polymorphisms and susceptibility of pre-eclampsia: a meta-analysis.,” *Ann. Hum. Genet.*, vol. 76, no. 5, pp. 377–86, Sep. 2012.

[39] S. Ni, Y. Zhang, Y. Deng, Y. Gong, J. Huang, Y. Bai, and R. Zhou, “AGT M235T polymorphism contributes to risk of preeclampsia: evidence from a meta-analysis.,” *J. Renin. Angiotensin. Aldosterone. Syst.*, vol. 13, no. 3, pp. 379–86, Sep. 2012.

[40] B. Dai, T. Liu, B. Zhang, X. Zhang, and Z. Wang, “The polymorphism for endothelial nitric oxide synthase gene, the level of nitric oxide and the risk for pre-eclampsia: a meta-analysis.,” *Gene*, vol. 519, no. 1, pp. 187–93, Apr. 2013.

[41] H.-P. Qi, W. D. Fraser, Z.-C. Luo, P. Julien, F. Audibert, and S.-Q. Wei, “Endothelial nitric oxide synthase gene polymorphisms and risk of preeclampsia.,” *Am. J. Perinatol.*, vol. 30, no. 10, pp. 795–804, Nov. 2013.

[42] H. Chen, G. Zhao, M. Sun, H. Wang, J. Liu, W. Gao, and T. Meng, “Endothelial nitric oxide synthase gene polymorphisms (G894T, 4b/a and T-786C) and preeclampsia: meta-analysis of 18 case-control studies.,” *DNA Cell Biol.*, vol. 31, no. 6, pp. 1136–45, Jun. 2012.

[43] Y. Li, M. Zhu, R. Hu, and W. Yan, “The effects of gene polymorphisms in angiotensin II receptors on pregnancy-induced hypertension and preeclampsia: a systematic review and meta-analysis.,” *Hypertens. pregnancy*, vol. 34, no. 2, pp. 241–60, May 2015.

[44] L. Zhao, A. T. Dewan, and M. B. Bracken, “Association of maternal AGTR1 polymorphisms and preeclampsia: a systematic review and meta-analysis.,” *J. Matern. Fetal. Neonatal Med.*, vol. 25, no. 12, pp. 2676–80, Dec. 2012.
