# Supplementary material for: Consensus strategy in genes prioritization and combined bioinformatics analysis for preeclampsia pathogenesis
Source: BMC Med Genomics. 2017 Aug 8;10:50. doi: 10.1186/s12920-017-0286-x (PMC5549357; doi:10.1186/s12920-017-0286-x)
Supplement: Supplementary file 5 — Integrated metabolic network. The file comprises the Integrated Metabolic Network corresponding with Model 3 of Table 8 as well as the list of all compounds contained in the metabolic network. (DOCX 1116 kb) [file 12920_2017_286_MOESM5_ESM.docx]

**Integrated Metabolic Network corresponding with Model 3 of Table 8.**


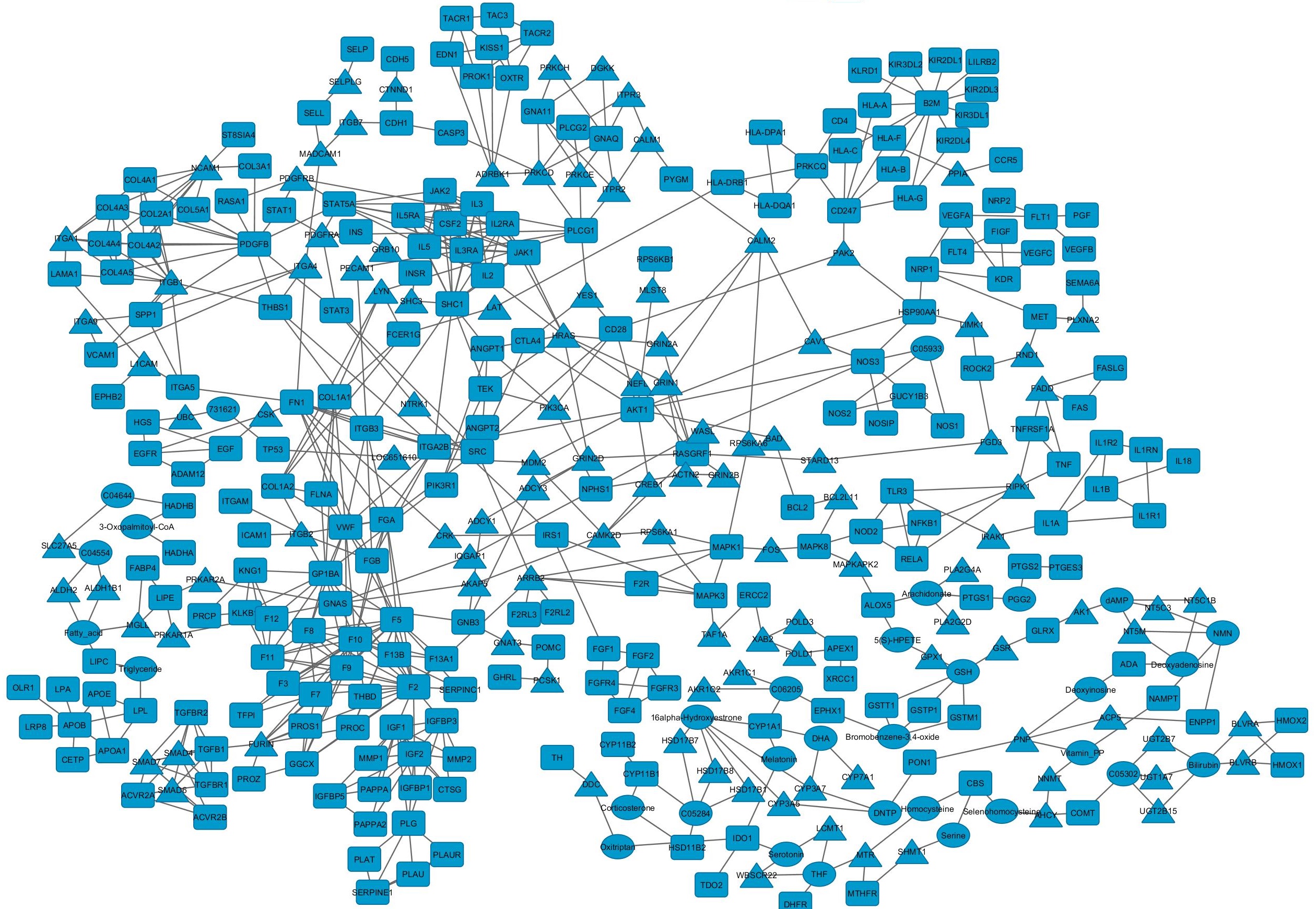


**Descriptions**

Ellipse: Compounds

Rectangles: input genes

Triangles: intermediate (missing) genes.

**Compounds List**

| 3-Oxopalmitoyl-CoA | C05259 | Deoxyadenosine | C00559 |
| --- | --- | --- | --- |
| Corticosterone | C02140 | Deoxyinosine | C05512 |
| Arachidonate | C00219 | Tetrahydrofolate (THF) | C00101 |
| 3alpha,7alpha-Dihydroxy-5beta-cholestanoyl-CoA | C04644 | Bromobenzene-3,4-oxide | C14839 |
| 11beta-Hydroxyandrost-4-ene-3,17-dione | C05284 | Parathion (DNTP) | C06604 |
| 3alpha,7alpha-Dihydroxy-5beta-cholestanate; | C04554 | Oxitriptan | C00643 |
| 2-Methoxy-17beta-estradiol | C05302 | Bilirubin | C00486 |
| Triglyceride | C00422 | Docosahexaenoic acid (DHA) | C06429 |
| 5(S)-HPETE | C05356 | Nicotinamide mononucleotide (NMN) | C00455 |
| 16alpha-Hydroxyestrone | C05300 | dAMP | C00360 |
| Prostaglandin G2 | C05956 | Melatonin | C01598 |
| L-Homocysteine | C00155 | Nicotinamide | C00153 |
| Glutathione (GSH) | C00051 | Serotonin | C00780 |
| Serine | C00065 | N(omega)-Hydroxyarginine; | C05933 |
